# 5-Hydroxymethylcytosine profiles of cfDNA are highly predictive of R-CHOP treatment response in diffuse large B cell lymphoma patients

**DOI:** 10.1186/s13148-020-00973-8

**Published:** 2021-02-11

**Authors:** Hang-Yu Chen, Wei-Long Zhang, Lei Zhang, Ping Yang, Fang Li, Ze-Ruo Yang, Jing Wang, Meng Pang, Yun Hong, Changjian Yan, Wei Li, Jia Liu, Nuo Xu, Long Chen, Xiu-Bing Xiao, Yan Qin, Xiao-Hui He, Hui Liu, Hai-Chuan Zhu, Chuan He, Jian Lin, Hong-Mei Jing

**Affiliations:** 1 Synthetic and Functional Biomolecules Center, Beijing National Laboratory for Molecular Sciences, Key Laboratory of Bioorganic Chemistry and Molecular Engineering of Ministry of Education, College of Chemistry and Molecular Engineering, Innovation Center for Genomics, Peking University, Beijing, 100871 People’s Republic of China; 2 Department of Hematology, Lymphoma Research Center, Peking University Third Hospital, Beijing, 100191 People’s Republic of China; 3 Lymphoma Head and Neck Oncology, Fifth Medical Center of PLA General Hospital, Beijing, 100039 People’s Republic of China; 4 Department of Medical Oncology, National Cancer Center/Cancer Hospital, Chinese Academy of Medical Sciences and Peking Union Medical College, Beijing, 100021 People’s Republic of China; 5 Department of Hematology, Beijing Hospital, National Center of Gerontology, Beijing, 1000730 People’s Republic of China; 6 Yang Sheng Tang Natural Medicine Research Institute, Hangzhou, 310024 People’s Republic of China; 7 Department of Chemistry, University of Chicago, Chicago, IL 60637 USA; 8 Institute of Biology and Medicine, College of Life and Health 20 Sciences, Wuhan University of Science and Technology, Hubei, 430081 People’s Republic of China

**Keywords:** Epigenetics, 5-Hydroxymethylcytosine (5hmC), Diffuse large B cell lymphoma, R-CHOP, Logistic regression modeling

## Abstract

**Background:**

Although R-CHOP (rituximab, cyclophosphamide, doxorubicin, vincristine, and prednisone) remains the standard chemotherapy regimen for diffuse large B cell lymphoma (DLBCL) patients, not all patients are responsive to the scheme, and there is no effective method to predict treatment response.

**Methods:**

We utilized 5hmC-Seal to generate genome-wide 5hmC profiles in plasma cell-free DNA (cfDNA) from 86 DLBCL patients before they received R-CHOP chemotherapy. To investigate the correlation between 5hmC modifications and curative effectiveness, we separated patients into training (*n* = 56) and validation (*n* = 30) cohorts and developed a 5hmC-based logistic regression model from the training cohort to predict the treatment response in the validation cohort.

**Results:**

In this study, we identified thirteen 5hmC markers associated with treatment response. The prediction performance of the logistic regression model, achieving 0.82 sensitivity and 0.75 specificity (AUC = 0.78), was superior to existing clinical indicators, such as LDH and stage.

**Conclusions:**

Our findings suggest that the 5hmC modifications in cfDNA at the time before R-CHOP treatment are associated with treatment response and that 5hmC-Seal may potentially serve as a clinical-applicable, minimally invasive approach to predict R-CHOP treatment response for DLBCL patients.

## Introduction

Diffuse large B cell lymphoma (DLBCL) is the primary type of invasive lymphoid tissue tumor, accounting for about 30% of non-Hodgkin's lymphoma [1]. Although the majority of the DLBCL patients are elderly patients, this disease is found in all ages [2]. Since rituximab (R) joined cyclophosphamide, adriamycin, vincristine, and prednisone (CHOP) chemotherapy regimen ten years ago, the overall survival rate of DLBCL patients has improved significantly [3].

However, 30–50% of patients are not sensitive to this standard treatment [4], and exiting methods fail to predict the treatment response before R-CHOP treatment accurately or efficiently [5, 6]. Currently, positron emission tomography (PET)-CT is the gold standard to evaluate the efficacy of different treatment regimens for DLBCL. However, it is generally used after the treatment and thus cannot predict the treatment response [7]. The International Prognostic Index (IPI) is the primary prognostic risk assessment method for DLBCL, especially in high-risk patients, and is used for R-CHOP chemotherapies [8–10]. However, IPI cannot accurately predict the therapeutic effect of R-CHOP in DLBCL patients [11]. Furthermore, recent studies have demonstrated that the detection of the apoptosis inhibitor, survivin [12], activation­induced cytidine deaminase (AID) [13], plasma miRNA [14], exosome miRNA [15], and genes polymorphism [16, 17], as well as the presence of CD3 and FoxP3 in the immune microenvironment [18], were all potential indicators of treatment efficacy in DLBCL patients. However, these predictors showed contradictory results that have not been well solved. Therefore, an accurate and effective method to predict the response of R-CHOP regimen is highly necessary.

In recent years, cell-free DNA (cfDNA) in the circulating blood, which carries genetic and epigenetic information from cells of origin, has emerged as a promising noninvasive approach for the diagnosis and prognosis in cancer [19]. 5-Methylcytosines (5mCs) of DNA is an important epigenetic feature that plays an important role in gene expression and cancer development [20]. Kristensen et al. [21] found that the methylation of *DAPK1* in cfDNA from patients with DLBCL can be used to assess the effect of R-CHOP treatment. In the human genome, 5-methylcytosines (5mCs) in cfDNA are dynamic and reversible [22, 23] and can be oxidized into 5-hydroxymethylcytosines (5hmCs) through the ten-eleven translocation (TET) enzymes in an active DNA-demethylation process [24, 25]. Therefore, 5hmC, as an oxidation product of DNA demethylation(5mC), may also be used to assess the effect of R-CHOP treatment. Recently, a study has also shown that 5hmC is associated with the prognosis of DLBCL [26]. However, its role in the prediction of treatment response of R-CHOP scheme for DLBCL patients is not established.

In this study, we used 5hmC-Seal technique to obtain genome-wide 5hmC profiles in plasma cfDNA from 86 DLBCL patients, before they received R-CHOP chemotherapy. Our results demonstrated that responders and non-responders of R-CHOP treatment had distinct 5hmC profiles and that 5hmC markers selected by bioinformatics tools and machine learning algorithms could be used to predict treatment response of R-CHOP treatment in DLBCL patients.

## Materials and methods

### Study participants

From 2017 to 2019, 86 diffuse large B cell lymphoma (DLBCL) patients from multicenter studies including Peking University Third Hospital, Fifth Medical Center of PLA General Hospital, and Cancer Hospital Chinese Academy of Medical Sciences were included in this study. All patients had signed the patient consent form. In all cases, the diagnosis of DLBCL was made using appropriate diagnostic criteria from the 2016 WHO classification of lymphoid tumors with combinations of histologic, immunohistochemical, and cell of origin (coo) defined according to the Hans algorithm [27]. Medical records were reviewed for demographic and clinical data. Laboratory tests, white blood cell count (WBC), renal and hepatic function examinations, lactate dehydrogenase (LDH), and β2 microglobulin (β2MG) and cfDNA from peripheral blood samples were collected before any treatment. Then, all patients received standard R-CHOP chemotherapy. Other baseline assessments including bone marrow biopsy and PET/CT were conducted in all patients in the follow-up care. The disease stage was defined by the Ann Arbor staging system. Treatment efficacy was evaluated after four cycles of treatment according to Lugano 2014 criteria [28], and patients were divided into PD (progressive disease), SD (stable disease), PR (partial response), and CR (complete response) based on the treatment outcome. This study was conducted in accordance with the Declaration of Helsinki.

### Study design

**Fig. 1:**
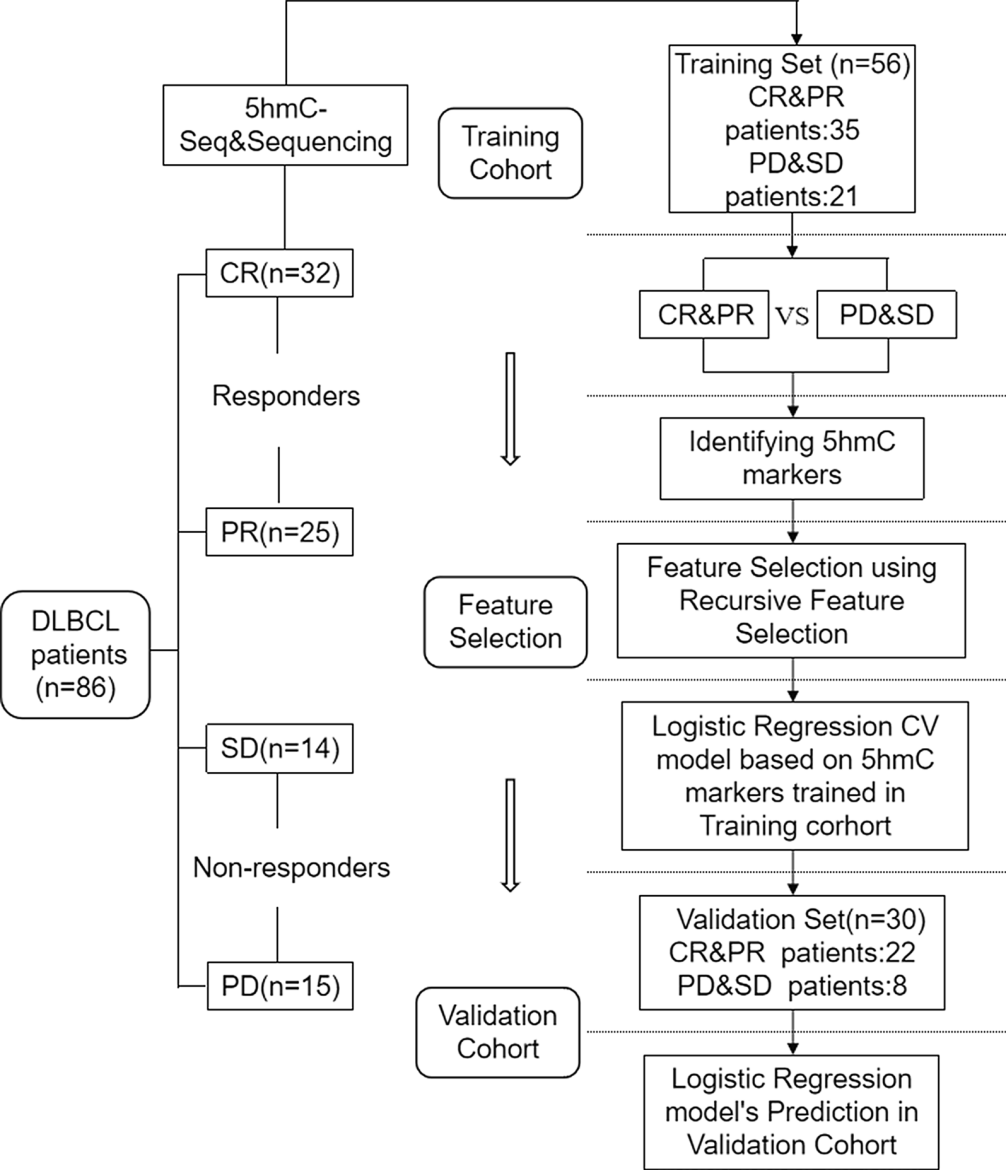
Overview of study design. A total of 86 cfDNA samples were collected at the time of diagnosis from patients with DLBCL before R-CHOP or R-CHOP-like treatment. Based on treatment outcome in the follow-up care, patients were divided into the responder group (PR & CR) and non-responder group (PD & SD). A logistic regression model was trained by the training cohort that was used to predict treatment response in the validation cohort

This study aimed to discover 5hmC markers to predict the curative effectiveness of R-CHOP scheme through high-efficiency hmC-Seal technology. Among the 86 patients recruited, PR and CR patients were grouped as responders (*n* = 57), and PD and SD patients were grouped as non-responders (*n* = 29) to R-CHOP treatment. We split 86 patients into a training and validation cohort. The objective of the first part of the study was to screen candidate genes with differential 5hmC modifications in these two groups from the training cohort. The objective of the second part of the study was to predict treatment outcome, using the model developed in the first part, in the validation cohort (Fig. 1).

### Clinical samples collection and cfDNA preparation

Eight milliliters of peripheral blood from DLBCL patients was collected into Cell-Free DNA Collection Tubes (Roche). Within 24 h, plasma was prepared by centrifuging twice at 1350×*g* for 12 min at 4 °C and 13,500×*g* for 12 min at 4 °C. Then, the plasma samples were immediately stored at – 80 °C. The plasma cfDNA was extracted using the Quick-cfDNA Serum & Plasma Kit (ZYMO) and then stored at − 80 °C. The fragment size of all the cfDNA samples was verified by nucleic acid electrophoresis before library preparation.

### 5hmC library construction and high-throughput sequencing

5hmC libraries for all samples were constructed with high-efficiency hmC-Seal technology [29]. Due to the highly sensitive nature of the chemical labeling method, the input cfDNA can be as low as 1–10 ng. According to the requirements of next-generation sequencing, the cfDNA extracted from plasma was end-repaired, 3′-adenylated using the KAPA Hyper Prep Kit (KAPA Biosystems), and then ligated with the Illumina compatible adapters. The ligated cfDNA was added in a glycosylation reaction in 25 μL solution containing 50 mM HEPES buffer (pH 8.0), 25 mM MgCl_2_, 100 μM UDP-6-N3-Glc, and 1 μM β-glucosyltransferase (NEB) for 2 h at 37 °C. Next, the cfDNA was purified using DNA Clean & Concentrator Kit (ZYMO). The purified DNA was incubated with 1 μL of DBCO-PEG4-biotin (Click Chemistry Tools, 4.5 mM stock in DMSO) for 2 h at 37 °C. Similarly, the DNA was purified using the DNA Clean & Concentrator Kit (ZYMO). Meantime, 2.5 μL streptavidin beads (Life Technologies) in 1 × buffer (5 mM Tris pH 7.5, 0.5 mM EDTA, 1 M NaCl, and 0.2% Tween 20) was added directly to the reaction for 30 min at room temperature. Finally, the beads were subsequently washed eight times for five minutes with buffer 1–4. All binding and washing steps were performed at room temperature with gentle rotation. Then, the beads were resuspended in RNase-free water and amplified with 14–16 cycles of PCR amplification. The PCR products were purified using AMPure XP beads (Beckman), according to the manufacturer’s instructions. The concentration of libraries was measured with a Qubit 3.0 fluorometer (Life Technologies). Paired-end 39-bp high-throughput sequencing was performed on the NextSeq 500 platform.

### Mapping and identifying 5hmC-enriched regions

FastQC (version 0.11.5) was used to assess the sequence quality. Raw reads were aligned to the human genome (version hg19) with bowtie2 (version 2.2.9) [30] and further filtered with SAMtools (version 1.3.1) [31], (parameters used: SAMtools view -f 2 -F 1548 -q 30 and SAMtools rmdup) to retain unique non-duplicate matches to the genome. Pair-end reads were extended and converted into BedGraph format normalized to the total number of aligned reads using bedtools (version 2.19.1) [32], and then converted to bigwig format, using bedGraphToBigWig from the UCSC Genome Browser for visualization in the Integrated Genomics Viewer. Potential 5hmC-enriched regions (hMRs) were identified using MACS (version 1.4.2), and the parameters used were macs 14 -p 1e-3 -f BAM -g hs [33]. Peak calls were merged using bedtools merge, and only those peak regions that appeared in more than 10 samples and that were less than 1000 bp were retained. Blacklisted genomic regions that tend to show artifact signals, according to ENCODE, were also filtered. The hMRs for each patient were generated by intersecting the individual peak call file with the merged peak file. The hMRs within chromosome X and Y were excluded and used as an input for the downstream analyses.

### Feature selection, model training, and validation

A two-step procedure was used to select optimal hMRs for distinguishing the non-responder group from the responder group prior to R-CHOP treatment. In step 1, DLBCL patients were randomly divided into training and validation cohorts in a stratified manner, using train_test_split in Scikit-Learn (version 0.22.1) [34] package in Python (version 3.6.10. In the training cohort, we identified differentially modified 5hmC regions (DhMRs) using EdgeR package (version 3.24.3) [35] in R (version 3.5.0), with the filtering threshold (*p* value < 0.01 & log2FoldChange > 0.5). In step 2, the dhMRs were further filtered using the recursive feature elimination algorithm (RFECV) in Scikit-Learn (parameters used: estimator = LogisticRegressionCV (class_weight = 'balanced', cv = 2, max_iter = 1000), scoring = 'accuracy').

Then, we trained the logistic regression CV model (LR) with the features selected from step 2 (parameter used: maxiter = 100, method = "lbfgs"). The trained LR model was used to predict the treatment outcome for patients in the validation cohort. Receiver operating characteristics (ROC) analysis was used to evaluate model performance. Area under the curve (AUC), best cutoff point, sensitivity, and specificity were computed with sklearn.metrics module.

### Exploring functional relevance of the 5hmC markers

We annotated the dhMRs from step 1 using the ChIPseeker package (version 1.20.0) [36], and genes that were closest to the marker regions were used for the following functional analyses. The GO enrichment analysis (Biological Process) was done by the ClueGO (version 2.5.5) and CluePedia (version 1.5.5) plug-in from Cytoscape software (version 3.7.2) (parameters used: medium network specificity, Bonferroni step-down pV correction and two-sided hypergeometric test). We used the Search Tool for the Retrieval of Interacting Genes (STRING) database (version 10.0, https://string-db.org) to find protein–protein interactions for 5hmC markers. Then, the Cytoscape software was used to construct the PPI network.

### Survival analysis and gene expression correlation analysis in TCGA-DLBC

For survival analysis, we downloaded the mRNA HTseq-FPKM data of 48 DLBLC patients from the TCGA-DLBC dataset [37] in the GDC Data Portal using gdc-client (version 1.5.0) and downloaded manually curated clinical data, including overall survival (OS), disease-specific survival (DSS), disease-free interval (DFI), and progression-free interval (PFI) from UCSC Xena [38]. Survminer package (version 0.4.6) and Surviva packages (version 2.44-1.1) in R were used for survival analysis. Forty-eight patients were divided into the high-expression group and low-expression group according to the cutoff points determined by the maximally selected rank statistics algorithm (maxstat) [39]. Survival analysis of each gene was assessed by Kaplan–Meier curves [40] and the log-rank test [41]. For the survival analysis, *p* value < 0.05 was considered statistically significant. For gene expression correlation analysis, we used a web tool called TIMER2.0 [42], which incorporated all TCGA expression data, to explore the mRNA expression relationship between 5hmC markers and other genes of interests in the TCGA-DLBC dataset. The correlation analysis was done using Spearman rank correlation.

### Statistical analysis

For clinical data, continuous variables are presented as mean (SD) and categorical variables are presented as count (percentages). To understand the relationship between categorical/continuous variables and treatment outcome, Kruskal–Wallis test by ranks [43] and *χ*^2^ test [44] were used, respectively. A two-sided *p* value of < 0.05 was considered to indicate statistical significance. The predictive power of clinical data was estimated by glm function in R-base and pROC package (version 1.15.3) in R.

## Results

### Clinical characteristics of Diffuse large B Cell lymphoma (DLBCL) patients

The clinical summary, including baseline characteristics and laboratory data, of all 86 patients is shown in Table 1. Of the 86 patients with DLBCL patients, 46 were male and 40 were female. The median age of all the patients was 54.6 years, and 63.9% of patients had advanced disease (including stage III and stage IV). Importantly, all patients were newly diagnosed with DLBCL and received standard R-CHOP chemotherapy. Treatment efficacy was evaluated in all patients after 4 cycles of treatment. According to the efficacy standard of Lugano 2014 criteria, the treatment response of patients was as follows: CR in 32 patients (37.2%), PR in 25 patients (29.1%), SD in 14 patients (16.3%), and PD in 15 patients (17.4%). Besides, according to the Hans model, 23 patients (26.7%) were germinal center B cell (GCB), 61 patients (70.9%) had non-GCB and 2 patients (2.3%) had an unknown cell of origin. The results of the international prognostic index (IPI) score showed that 52.3% of patients (IPI score > 2) belonged to the high–intermediate-risk/high-risk group. Finally, the mean of WBC, LDH, and β2MG for all patients was 6.94 × 10–4/L, 364.33 U/L, and 2.84 mg/L, respectively.

**Table 1:** Diffuse large B cell lymphoma (DLBCL) patient characteristics

Characteristics	Level/type	Value
*n*		86
Sex (%)	F	40 (46.5)
	M	46 (53.5)
Age (mean (SD))		54.59 (15.56)
Diagnosis (%)	DLBCL	86 (100.0)
Therapy (%)	R-CHOP	86 (100.0)
Response (%)	CR	32 (37.2)
	PD	15 (17.4)
	PR	25 (29.1)
	SD	14 (16.3)
Ann Arbor stage (%)	I	6 (7.0)
	II	18 (20.9)
	III	7 (8.1)
	IV	48 (55.8)
	Unknown	7 (8.1)
Cell of origin (%)	GCB	23 (26.7)
	Non-GCB	61 (70.9)
	Unknown	2 (2.3)
IPI (%)	0	8 (9.3)
	1	12 (14.0)
	2	18 (20.9)
	3	28 (32.6)
	4	15 (17.4)
	5	2 (2.3)
	Unknown	3 (3.5)
Mean LDH (SD)		364.33 (326.72)
Mean β2MG (SD)		2.84 (2.77)

IPI, International Prognostic Index; GCB, germinal center B cell; CR, complete response; PR, partial response; SD, stable disease; PD, progressive disease; LDH, lactate dehydrogenase; β2MG, beta2 microglobulin; WBC, white blood cell

### 5hmC profiles differ between responders and non-responders to R-CHOP treatment in the training cohort

**Fig. 2:**
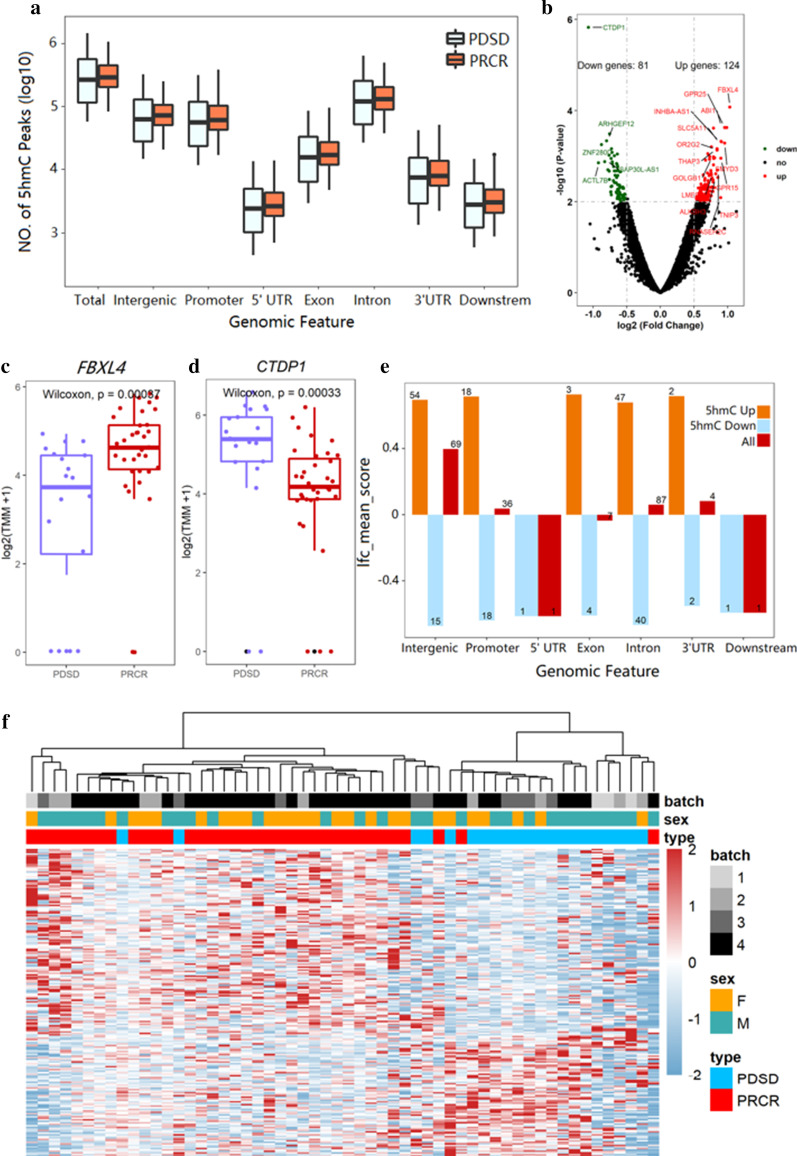
Characteristics of 5hmC distribution in plasma cfDNA of DLBCL patients in the training cohort (*n* = 56). **a** Genome-wide 5hmC distribution in different genomic features grouped by R-CHOP treatment response (PDSD vs PRCR). **b** Volcano plot. Significantly altered genes (abs (log2 Foldchange) ≥ 0.5; *p* value < 0.01) are highlighted in red (up) or green (down) using the responder group (PRCR) as the reference (*n* = 205). Black dots represent the genes that are not differentially expressed. **c**, **d** Boxplots of *FBXL4* and *CTDP1* grouped by treatment response (PDSD vs PRCR). Log2 transformed of TMM normalized 5hmC enrichment values were plotted, and the Wilcoxon *t *test was used. **e** Mean log2 Foldchange value of 205 DhMRs across different genomic features (Orange for 124 5hmC-up DhMRs, blue for 81 5hmC-down DhMRs, red for all 205 DhMRs). **f** Heatmap of 205 DhMRs markers with treatment response, batch, and sex information labeled. Unsupervised hierarchical clustering was performed across genes and samples

Eighty-six DLBCL patients were randomly divided into the training cohort (n = 56) and validation cohort (*n* = 30) (Fig. 1). We used hmC-Seal to generate genome-wide 5hmC profiles for patients in the training set, including 35 responders and 21 non-responders to R-CHOP treatment. The overall 5hmC enrichment (all hMRs) was most common in intronic, intergenic, and promoter regions for both responders and non-responders, even though no statistically significant difference was found between these two groups for any genomic feature types (Fig. 2a). Meanwhile, we conducted differential analysis (EdgeR; *p* < 0.01, fold change > 0.5) and observed 205 DhMRs, including upregulate (*n* = 124) and downregulate (n = 81) regions in responders compared to non-responders (Fig. 2b). For instance, *FBXL4* (Fig. 2c) was highly enriched in hydroxymethylation for responders (*p* = 0.00087), and *CTDP1* (Fig. 2d) was highly enriched in hydroxymethylation for non-responders (*p* = 0.00033). In addition, for the top 205 DhMRs, the most significant enrichment was found in intronic, intergenic, and promoter regions, consistent with previous studies [45, 46] (Fig. 2e). Finally, heatmap results, using default clustering methods, demonstrated that these 205 DhMRs could effectively separate responders from non-responders (Fig. 2f).

### Pathway analysis and function exploration

**Fig. 3:**
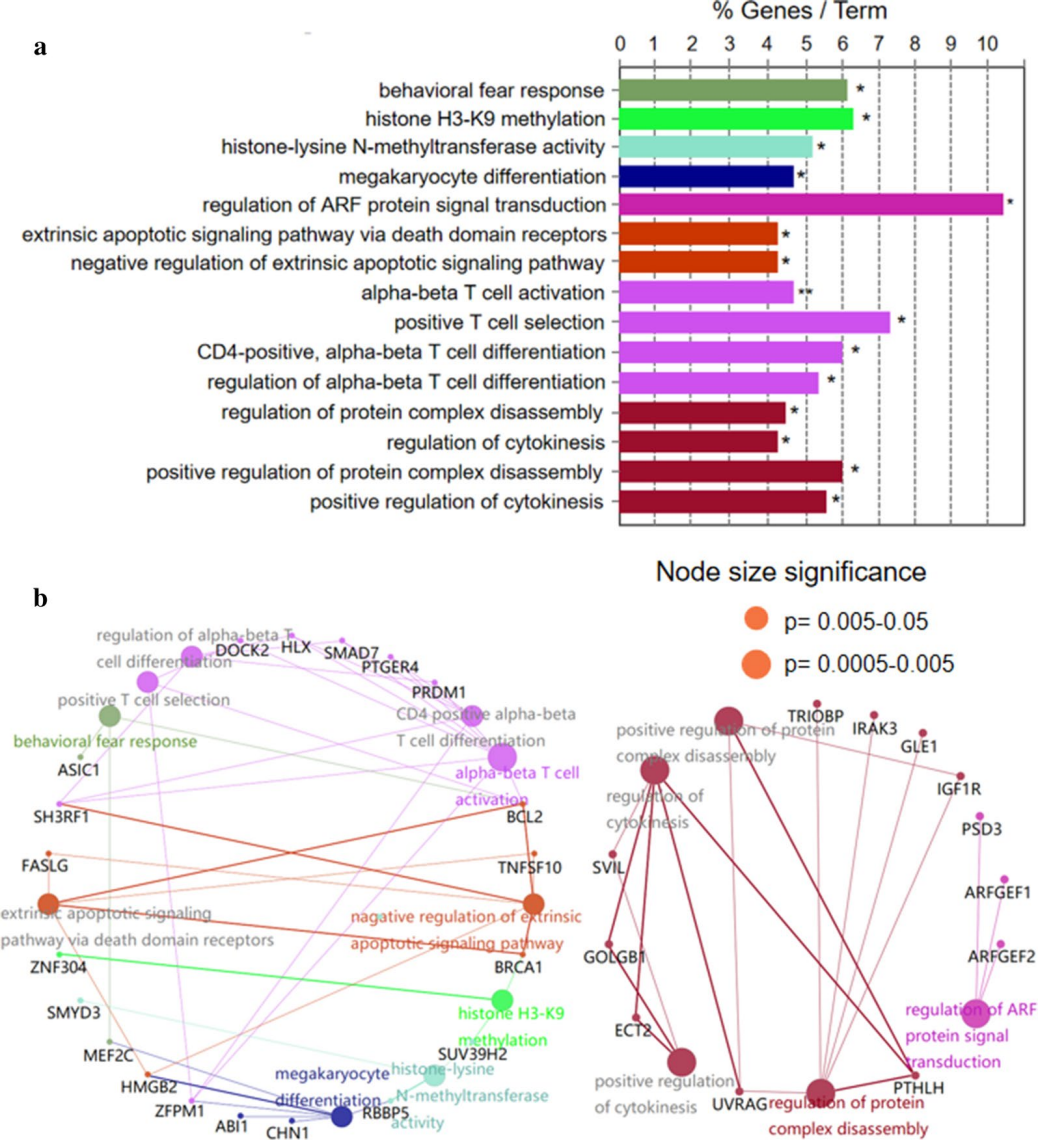
GO enrichment analysis and function exploration of 205 5hmC markers using Cytoscape software. **a** GO enrichment bar plot (*p = 0.005–0.05, **p = 0.0005–0.005). **b** GO enrichment and Gene-Concept Network. The node size is proportional to the *p* value calculated from the network

Pathway analysis of 205 5hmC markers (Additional file 1: Table 1) in DLBCL patients suggested functional enrichment in certain canonical pathways. The top enriched GO biological pathways included signaling like alpha–beta T cell differentiation, protein-lysine N-methyltransferase activity, and histone H3-K9 modification (Fig. 3a). Among these pathways, signaling by alpha–beta T cell differentiation was known to be relevant to tumor growth and apoptosis, which suggested that the DhMRs might be involved in the immunity system [47–49]. Meanwhile, the hubs of the GO functional interaction networks (Fig. 3b) showed that these genes, including BCL2 apoptosis regulator (*BCL2*), PR/SET domain 1 (*PRDM1*), prostaglandin E receptor 4 (*PTGER4*), SMAD family member 7 (*SMAD7*), H2.0 like homeobox (*HLX*), dedicator of cytokinesis 2 (*DOCK2*) and SH3 domain containing ring finger 1 *(SH3RF1),* participated in the regulating T cell activation and differentiation pathway.

### 5hmC markers showed prediction performance superior to clinical indicators for R-CHOP treatment response

**Fig. 4.**
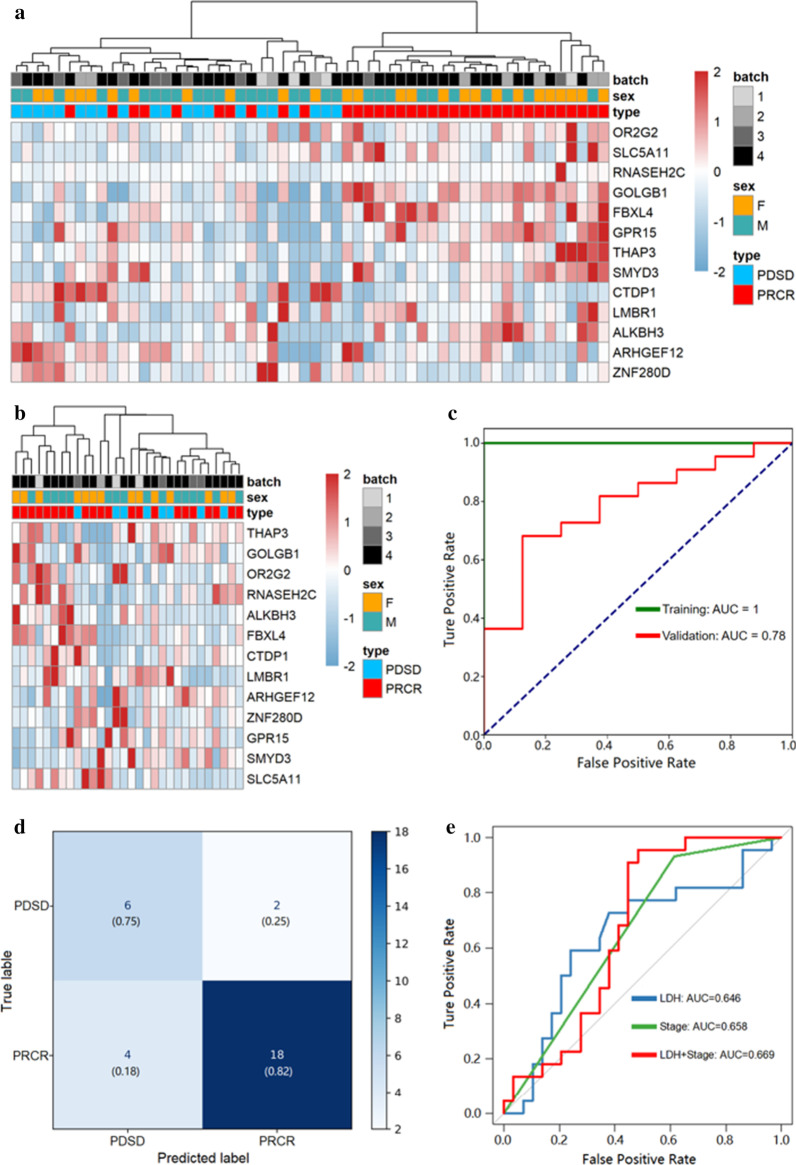
5hmC markers’ prediction for treatment response in the training and validation cohort. **a**, **b** Heatmaps of 13 5hmC markers with treatment response, batch and sex information labeled in the training and validation cohorts. Unsupervised hierarchical clustering was performed across genes and samples. **c** Receiver operating characteristic (ROC) curve of the classification model with 13 5hmC markers in the training and validation cohorts. The true-positive rate (sensitivity) is plotted in function of the false-positive rate (1-specificity). **d** Confusion matrix that shows the model performance in the validation cohort (responders: 22, non-responders:8). **e** ROC curve of the classification model with LDH, stage and LDH combined with stage for DLBCL patients

Similarly, we generated genome-wide 5hmC profiles for patients in the validation set, including 22 responders and 8 non-responders to R-CHOP treatment. By using the recursive feature elimination algorithm based on the logistic regression CV estimator, we further reduced the number of 5hmC markers from 205 to 13, which achieved the best cross-validation score (Additional file 2: Figure S1). Further, we found that the 13 5hmC markers (Table 2), selected by the LR model, could distinguish responders from non-responders in both the training and validation cohorts (Fig. 4a, b). Meantime, these 13 5hmC markers could effectively predict responders and non-responders to R-CHOP treatment in the training (AUC = 1.00) and the validation cohorts (AUC = 0.78) (Fig. 4c), achieving 0.82 sensitivity and 0.75 specificity in the validation cohort (Fig. 4d). Finally, we also calculated the individual AUC for each of the 13 5hmC markers in the training and validation cohorts (Additional file 2: Figure S2A, B). Among these, *ARHGEF12* and *ZNF280D* showed the best predictive performance, yielding an AUC of 0.76 in the validation cohort.

**Table 2:** Coefficients for 13 5hmC markers in the logistic regression model trained by the training cohort

Markers	GeneID	Coefficients	SE	*z* value	*p* value
Intercept		− 5.5704	0.867	2.652	< 0.01
chr1_6721489_6721898	THAP3	0.7712	0.145	1.865	< 0.05
chr1_246290825_246291238	SMYD3	0.39	0.149	1.955	< 0.05
chr1_247755954_247756505	OR2G2	3.1779	0.108	1.344	< 0.05
chr11_43905400_43905804	ALKBH3	3.3423	0.128	1.306	< 0.05
chr11_65511519_65512429	RNASEH2C	1.5211	0.072	2.061	< 0.05
chr11_120211662_120212234	ARHGEF12	− 3.8797	0.115	− 3.225	< 0.001
chr15_56982146_56982638	ZNF280D	− 1.2266	0.177	− 3.250	< 0.001
chr16_24916341_24916920	SLC5A11	0.6683	0.076	0.149	< 0.05
chr18_77500908_77501376	CTDP1	− 2.573	0.103	− 3.182	< 0.001
chr3_98270705_98271079	GPR15	0.1052	0.167	2.348	< 0.05
chr3_121430838_121431239	GOLGB1	0.8526	0.178	2.982	< 0.01
chr6_99461404_99461922	FBXL4	1.7188	0.101	2.165	< 0.05
chr7_156700537_156701031	LMBR1	1.0942	0.078	0.579	< 0.05

SE, standard errors of coefficients; *z* value, Wald *z*-statistic value

We also investigated the association between available clinical indicators, including stage, pathology, IPI, LDH, β2MG and WBC, and R-CHOP treatment response. Among all those clinical indicators, only LDH (continuous variable, *p* = 0.03474) and stage (categorical variable, *p* = 0.004453) showed a significant association with treatment response (Additional file 2: Table 3). Thus, we used these two indicators to build logistic regression models to predict treatment response. As expected, LDH level, stage, and LDH combined with stage (LDH + stage) could also predict treatment response to a certain level. However, the AUC of LDH level (AUC = 0.646), stage (AUC = 0.658), and LDH combined with stage (AUC = 0.669) were lower than that of 5hmC markers (AUC = 0.78) (Fig. 4e).

### Potential associations between 5hmC markers and R-CHOP treatment response in DLBCL patients

**Fig. 5:**
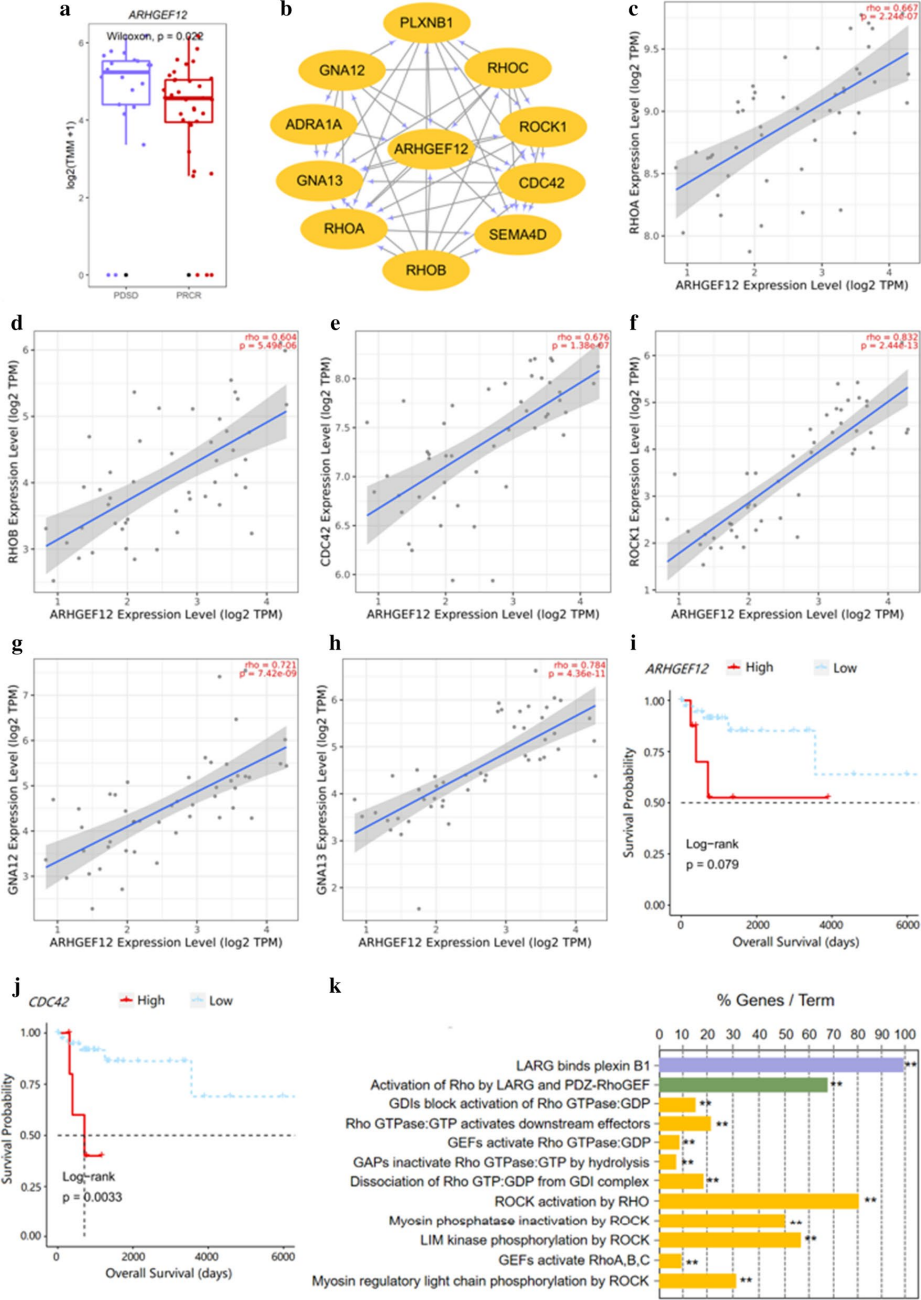
*ARHGEF12* and its potential relevance in DLBCL patients and treatment response. **a** Boxplot of *ARHGEF12* grouped by treatment response (PDSD vs PRCR). Log2 transformed of TMM normalized 5hmC enrichment values were plotted, and Wilcoxon *t *test was used. **b** Functional protein–protein interaction networks (PPI) from the STRING database. **c**–**h** Correlation plots of the mRNA expression of *ARHGEF12* with the mRNA expressions of genes in the RHO pathway, including *RHOA*, *RHOB*, *CDC42*, *ROCK1*, *GNA12* and *GNA13* in DLBCL in the TCGA-DLBC dataset. **i**, **j** Overall survival curves of DLBCL patients with low or high gene expressions in *ARHGEF12* or *CDC42* in the TCGA-DLBC dataset. The *x*-axis represents the OS time (days), and the *y*-axis represents the survival probability. (K) GO enrichment bar plot for genes associated with *ARHGEF12* as shown in the PPI network (*p = 0.005–0.05, **p = 0.0005–0.005)

To further understand the potential associations between those 13 5hmC-modified marker genes and R-CHOP treatment response, we investigated their mRNA expression profiles and compared them to that of B-lymphocyte antigen CD20 (*MS4A1*), a rituximab target gene, in 48 DLBCL patients from the TCGA-DLBC dataset. Among those 13 marker genes, we found that the mRNA expression of *MS4A1* was positively correlated with the mRNA expression of *ARHGEF12* (rho = 0.385), *FBXL4* (rho = 0.376), *GOLGB1* (rho = 0.434*)*, *LMBR1* (rho = 0.45) (Additional file 2 Figure S3A–D). We decided to further investigate the potential mechanism of Rho Guanine Nucleotide Exchange Factor 12 (*ARHGEF12*), for its mRNA expression was positively associated with *MS4A1*, and it achieved the highest AUC in the validation cohort among 13 5hmC-modified marker genes. According to recent studies, 5hmC enrichment in promoter regions was positively associated with gene expression levels [25, 50]. In our study, *ARHGEF12* was highly enriched in hydroxymethylation in the non-responders (*p* = 0.022) (Fig. 5a), and the hydroxymethylation site was in the promoter region (Additional file 3: Table 2). Therefore, we speculated that the change in 5hmC enrichment in the promoter region of *ARHGEF12* might lead to the change in the mRNA expression of this gene.

In addition, from the PPI network constructed from the STRING database, we identified several genes linked to *ARHGEF12*, including Ras Homolog Family Member A (*RHOA*), Ras Homolog Family Member B (*RHOB*), Ras Homolog Family Member C (*RHOC*), Cell Division Cycle 42 (*CDC42*), Rho Associated Coiled-Coil Containing Protein Kinase 1 (*ROCK1*), G Protein Subunit Alpha 12 (*GNA12*) and G Protein Subunit Alpha 13 (*GNA13*) (Fig. 5b). Interestingly, we found that all of these gene expressions (*RHOA* (rho = 0.667), *RHOB* (rho = 0.604), *CDC42* (rho = 0.676), *ROCK1 *(rho = 0.832)*, GNA12* (rho = 0.721), GNA13 (rho = 0.784)) were highly positively associated with that of *ARHGEF12* (Fig. 5c–h)*.* Moreover, from survival analysis results in the TCGA-DLBC dataset, we found that the overall survival time (OS, days) of patients with high expression of *ARHGEF12* and *CDC42* was significantly lower than that of patients with low expression in these 2 genes (Fig. 5i, j). Also, we found that the mRNA expression of *ARHGEF12* was positively associated with several immune-related genes, such as *CD44*, *CD47*, *CD53*, *CD59*, and *CD274* (Additional file 2: Figure S4A–E). Finally, we conducted a GO enrichment analysis (Fig. 5k) for all the genes associated with *ARHGEF12* (Fig. 5b) and found that the main GO enrichment was in the Rho signaling pathway which was consistent with the PPI network constructed from the STRING database.

## Discussion

Even though previous studies have reported that 5hmC modifications could serve as potential diagnostic and prognostic markers in DLBCL patients [26], its role in the prediction of treatment response of R-CHOP scheme was not fully studied. Therefore, an accurate, noninvasive prediction test for treatment response of R-CHOP is highly desirable, and to this end, the emergence of liquid biopsy technology has shown to be a promising approach. In this study, we aimed to develop a model to predict R-CHOP scheme treatment response for DLBCL patients based on the 5hmC profiles derived from plasma cfDNA before R-CHOP treatment using hmC-Seal sequencing method.

In our cohort, we found that responders and non-responders to R-CHOP scheme had distinctive differences in 5hmC enrichment, containing 205 DhMRs detected by differential analysis method. Additionally, pathway analysis of the 205 marker genes with differentially modified 5hmC between responders and non-responders suggested enrichment in alpha–beta T cell activation and differentiation signaling pathway. As we all known, tumor progression and drug resistance are highly associated with the physiological state of the tumor microenvironment (TME), and thus, the tumor microenvironment (TME) represents an attractive therapeutic target and closely related to the curative effect of tumor therapy [47]. The composition of tumor microenvironment is complex, which mainly include tumor cells, stromal elements, extracellular matrixes, inflammation, and immune cells [48], which are closely related to tumor development, metastasis, and tumor therapy [49]. Importantly, cfDNA is not only derived from tumor cells, but also from the tumor microenvironment [51]. Therefore, these 5hmC marker genes could be related to the effect of R-CHOP treatment.

Furthermore, we found that 13 5hmC markers filtered by machine learning algorithms could well distinguish non-responders from responders in both the training and validation cohorts. Meantime, the prediction performance of the logistic regression model, established by 13 5hmC markers, achieving 0.82 sensitivity and 0.75 specificity (AUC = 0.78), was superior to existing clinical indicators, such as LDH (AUC = 0.646) and stage (AUC = 0.658). Furthermore, when combining the LDH and stage, the AUC was also lower than 13 5hmC markers. Taken together, these findings indicated that 5hmC markers derived from cfDNA may serve as effective biomarkers for minimally noninvasive prediction for treatment response of DLBCL patients with R-CHOP scheme.

According to recent studies, 5hmC enrichment in promoter regions can promote gene transcription [25]. In our study, the hydroxymethylation of *ARHGEF12* is enriched in the promoter region in non-responders. Notably, among 13 5hmC marker genes, *ARHGEF12* showed the best predictive performance, and its mRNA expression was positively associated with that of *MS4A1* in the TCGA-DLBC dataset. Meantime, *ARHGEF12* expression was highly positively correlated with Rho-related genes, such as *RHOA*, *RHOB*, *CDC42*, *ROCK1*, *GNA12*, and *GNA13*. Previous research suggested that Rho signaling pathway was linked to cancer microenvironment, cancer initiation, proliferation, and metastasis, and might incorporate novel biological implications and therapeutic opportunities [52]. These genes related to *ARHGEF12* all play crucial roles in the Rho signaling pathway, and their functions in cancer initiation, proliferation, metastasis, and drug resistance are well supported by previous research [52–57]. In addition, the potential functions of *ARHGEF12* were also reported in various researches. For instance, *ARHGEF12* is a well-studied activator of Rho signaling downstream of G-protein-coupled receptors (GPCRs) and has essential roles in chemokine-driven tumor cell invasion [58, 59]. More importantly, *ARHGEF12* expression was also positively associated with immune-related genes, such as *CD44*, *CD47*, *CD53*, *CD59*, and *CD274.* Therefore, we suspected that, like its related genes, *ARHGEF12* was crucial in the Rho signaling pathway and might be related to diffuse large B cell lymphoma initiation, proliferation, metastasis and treatment, but the deep biological basis of these relationships needs further study. Taken together, the above evidence provided by previous research suggested that *ARHGEF12* might serve as a potential drug target that was related to the treatment response of R-CHOP treatment.

Nevertheless, this study still has some limitations. First, the sample size is relatively small and may not fully represent all DLBCL patients. The performance of our model still needs to be tested in larger study cohorts. Second, this study only focuses on Chinese patients and may not represent DLBCL patients in other races. Thirdly, the regulatory mechanism of 5hmC in *ARHGEF12* and its relevance in R-CHOP treatment effectiveness are still not clear. Thus, further studies are required. In the future, we aim to increase the sample size of DLBCL patients and find more stable and reliable 5hmC marker genes to predict the treatment response of R-CHOP scheme.

In conclusion, our results suggested that 5hmC markers derived from plasma cfDNA can be used to predict treatment response of DLBCL patients treated with R-CHOP scheme. Meanwhile, hmC-Seal might serve as a minimally noninvasive technique to unveil potential drug targets related to the treatment response of R-CHOP in DLBCL patients.

## Supplementary information

**Additional file 1.** 205 5hmC markers.anotation.

**Additional file 2.** Supplementary material.

**Additional file 3.** 13 5hmC feature markers.anotation.

## Data Availability

The datasets supporting the conclusions of this article are included within the article and its additional files. All other datasets used and analyzed during the current study are available from the corresponding author on reasonable request.

## Abbreviations

R-CHOP: Rituximab, cyclophosphamide, doxorubicin, vincristine, and prednisone
DLBCL: Diffuse large B cell lymphoma
IPI: International Prognostic Index
AID: Activation­induced cytidine deaminase
5hmC: 5-Hydroxymethylcytosine
5mC: 5-Methylcytosine
TET: Ten-eleven translocation
cfDNA: Cell-free DNA
WBC: White blood cell count
LDH: Lactate dehydrogenase
β2MG: β2 Microglobulin
PD: Progressive disease
SD: Stable disease
PR: Partial response
CR: Complete response
hMRs: 5HmC-enriched regions
RFECV: Recursive feature elimination algorithm
ROC: Receiver operating characteristic
AUC: The area under ROC curves
STRING: Search Tool for the Retrieval of Interacting Genes
OS: Overall survival
DSS: Disease-specific survival
DFI: Disease-free interval
PFI: Progression-free interval
