# Supplementary material for: 5-Hydroxymethylcytosine profiles of cfDNA are highly predictive of R-CHOP treatment response in diffuse large B cell lymphoma patients
Source: Clin Epigenetics. 2021 Feb 11;13:33. doi: 10.1186/s13148-020-00973-8 (PMC7879534; doi:10.1186/s13148-020-00973-8)
Supplement: Supplementary file 2 — Additional file 2. Supplementary material. [file 13148_2020_973_MOESM2_ESM.doc]

1. **hydroxymethylcytosine Profiles of cfDNA are Highly Predictive of R-CHOP treatment Response in Diffuse Large B-Cell Lymphoma Patients**

Hang-Yu Chen†^1^, Wei-Long Zhang†^2^, Lei Zhang^1^, Ping Yang^2^, Fang Li^2^, Ze-Ruo Yang^6^, Jing Wang^2^, Meng Pang^2^, Yun Hong^2^, Changjian Yan^2^, Wei Li^2^, Jia Liu^2^, Nuo Xu^1^, Long Chen^1^, Xiu-Bing Xiao^3^, Yan Qin^4^, Xiao-Hui He^4^, Hui Liu^5^, Chuan He*^1^， Jian Lin*^1^, Hong-Mei Jing*^2^

Supplementary Information:


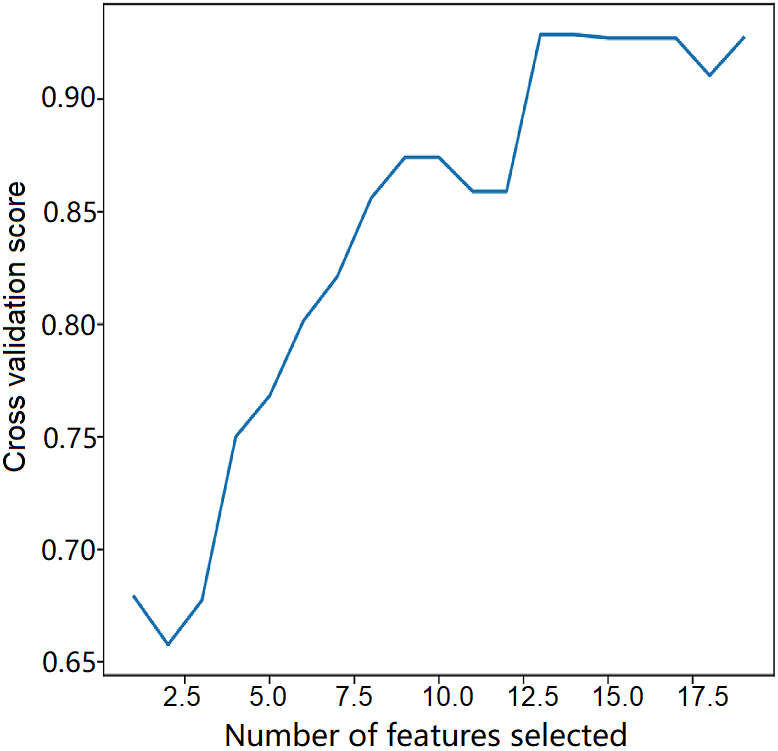


**Figure S1. Feature Selection for 5hmC markers.** The recursive feature selection algorithm selects 13 as the smallest number of features that achieve the best cross-validation score. The x-axis is the number of features selected and the y-axis is the cross-validation score for model performance.


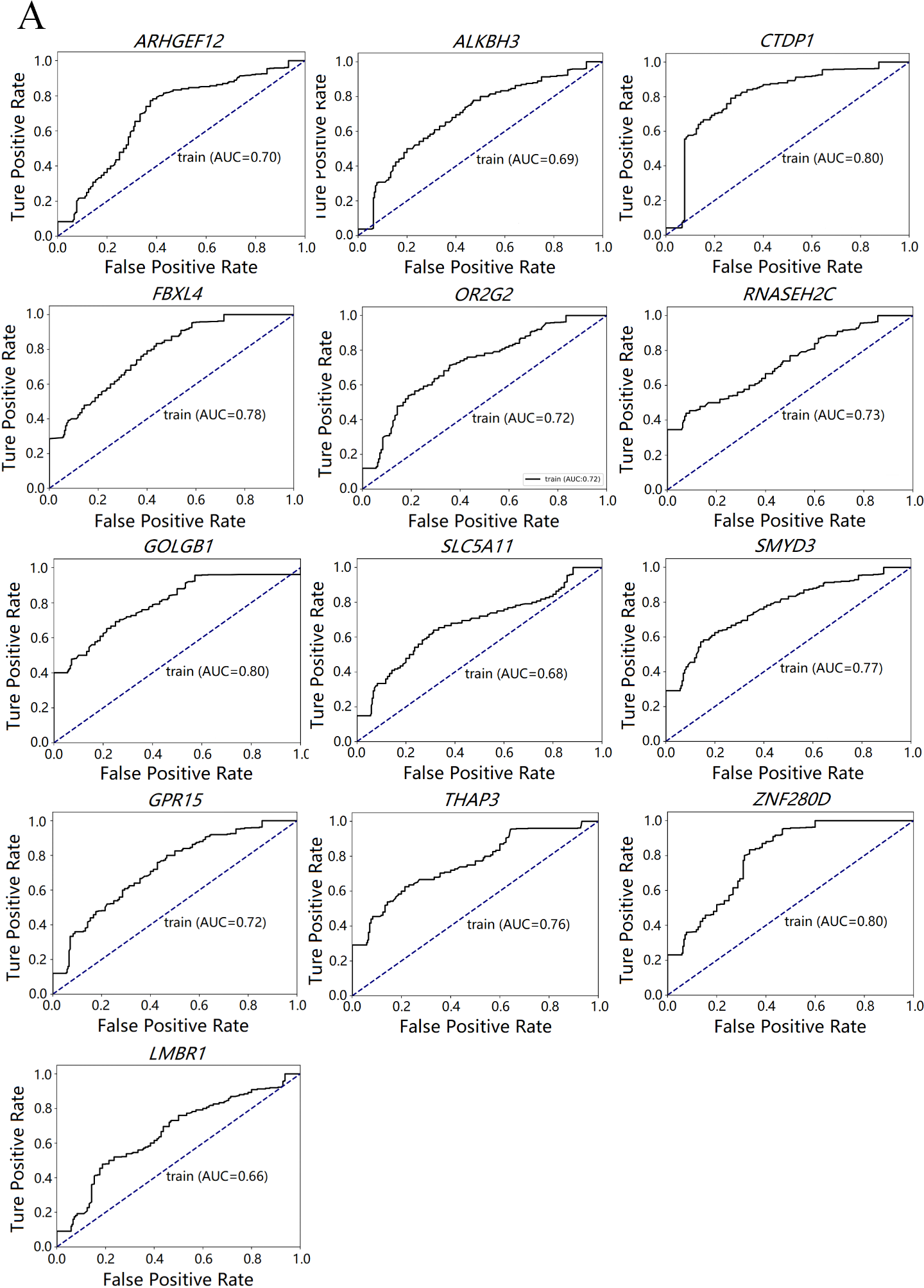


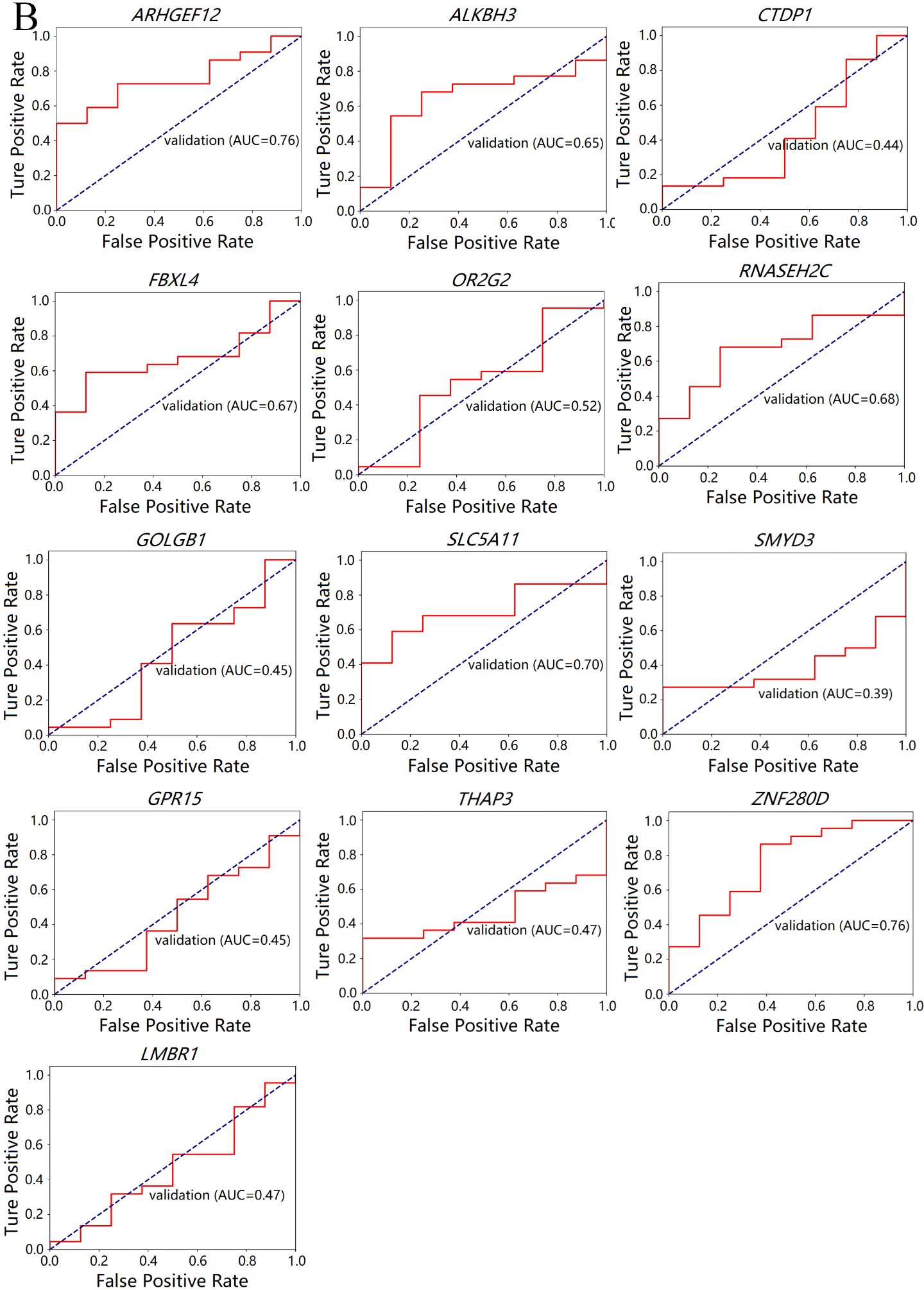


**Figure S2. The individual prediction performance of each marker in the thirteen markers in the training and validation cohort.** (A) Individual ROC curves for each marker in the training cohort. (B) Individual ROC curves for each marker in the validation cohort.


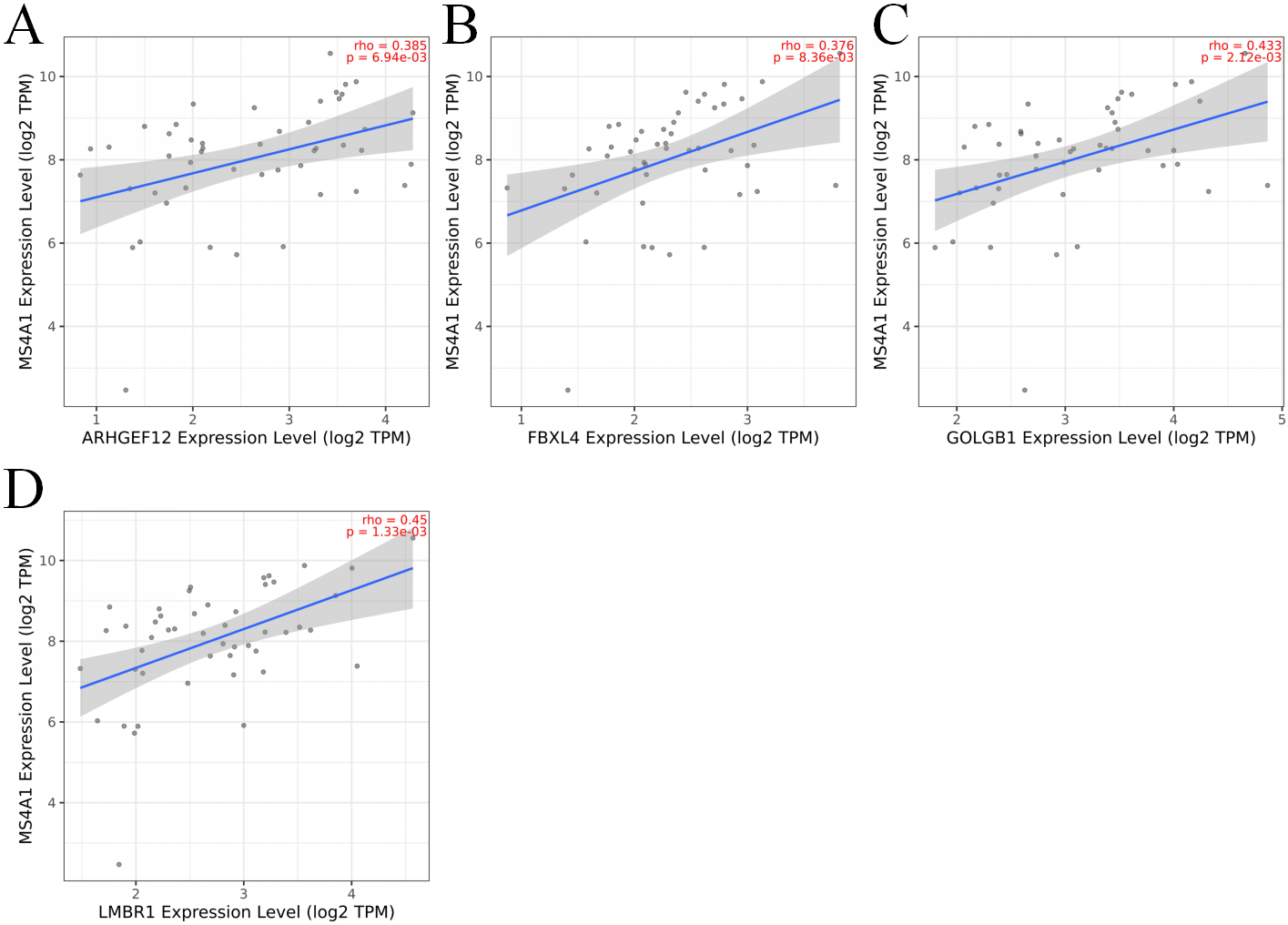


**Figure S3. mRNA expression correlation plot of *MS4A1* and 5hmC markers in the TCGA-DLBC dataset.** (A) *ARHGEF12*. (B) *FBXL4*. (C) *GOLGB1*. (D) *LMBR1*.


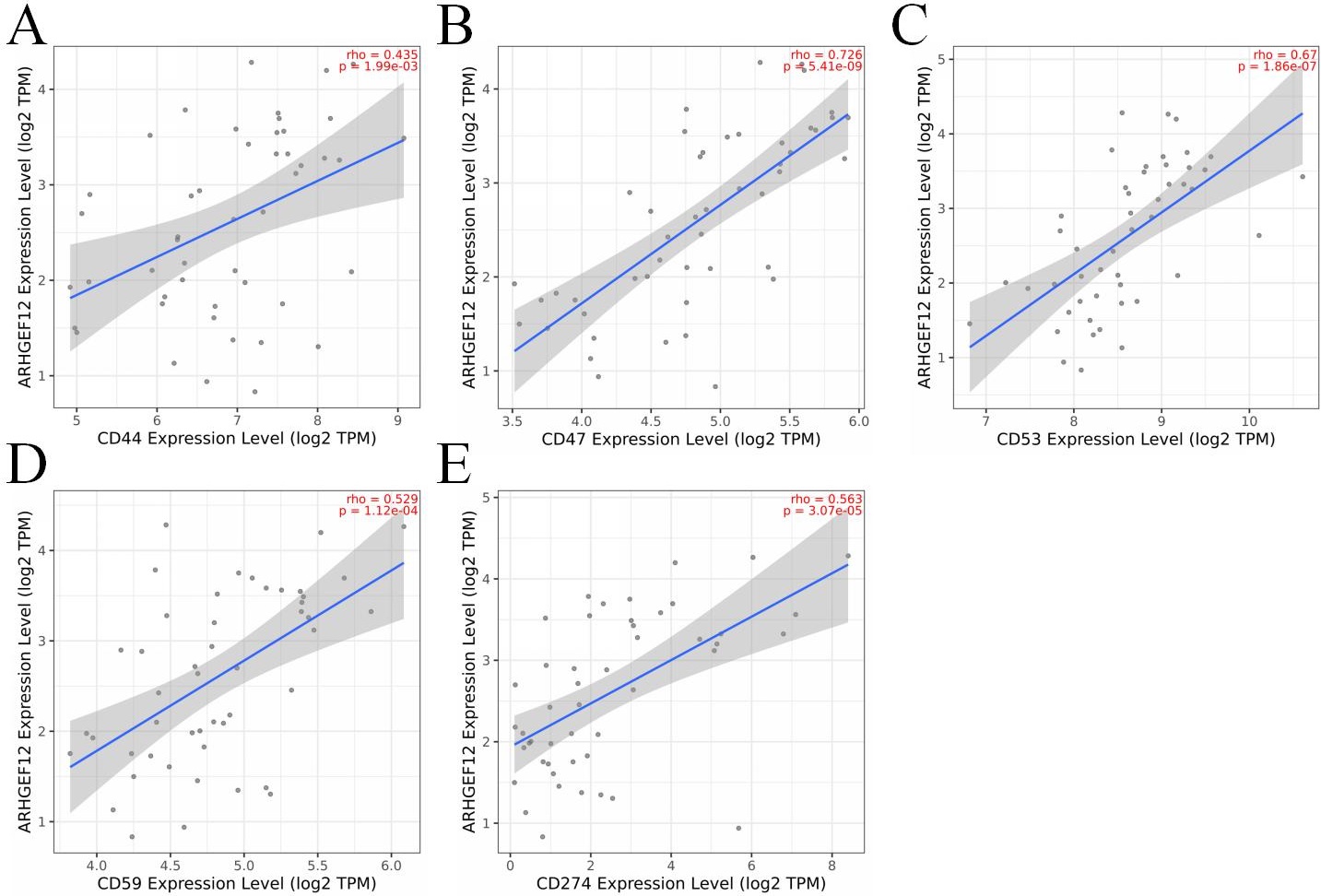


**Figure S4. mRNA expression correlation of *ARHGEF12* and immune-related genes in the TCGA-DLBC dataset.** (A) *CD44*. (B) *CD47*. (C) *CD53*. (D) *CD59*. (E) *CD274*.

Supplementary materials Table Table 3: Clinical indicators and their associations with treatment response.

| **Clinical indicators** | **chi-square statistic** | **Kruskal-Wallis chi-squared** | **df** | **p value** |
| --- | --- | --- | --- | --- |
| stage | 8.0893 |  | 1 | 0.004453 |
| Pathology | 0.30099 |  | 1 | 0.5833 |
| IPI | 2.4113 |  | 1 | 0.1205 |
| LDH |  | 4.458 | 1 | 0.03474 |
| β2MG |  | 2.8799 | 1 | 0.08969 |
| WBC |  | 2.5528 | 1 | 0.1101 |
